# Life-history data of a key amphipod species from three NE Atlantic estuaries under different levels of anthropogenic pressure

**DOI:** 10.1016/j.dib.2021.107729

**Published:** 2021-12-16

**Authors:** I. Martins, A. Guerra, N. Leite, E. Constantino, M.I. Ilarri, A.T. Souza, M.M. Santos, A.T. Ford, J. Campos

**Affiliations:** a MARE- Marine and Environmental Sciences Centre, Faculdade de Ciências e Tecnologia, Universidade de Coimbra, Coimbra 3004-517, Portugal; b SEAentia - Science Based Aquaculture, Parque Tecnológico de Cantanhede, Núcleo 04, Lote 2, Cantanhede 3060-197, Portugal; c CIIMAR- Interdisciplinary Centre of Marine and Environmental Research, University of Porto, Edifício do Terminal de Cruzeiros do Porto de Leixões, Avenida General Norton de Matos, s/n, Matosinhos 4450-208, Portugal; d Institute of Hydrobiology, Biology Centre of the Czech Academy of Sciences, Na Sádkách 7, 370 05 České Budějovice, Czech Republic; e FCUP – Department of Biology, Faculty of Sciences, University of Porto, Porto, Portugal; f School of Biological Sciences, Faculty of Sciences, University of Portsmouth, Institute of Marine Sciences Laboratories, Langstone Harbour, Ferry Road, Eastney, Portsmouth P04 9LY, United Kingdom

**Keywords:** *Echinogammarus marinus*, Intra-site habitats, Organic matter, *Fucus* spp., Population dynamics

## Abstract

Knowledge on population dynamics of ecosystem's key-species is invaluable to understand how populations will respond to natural and human-induced perturbations. The amphipod *Echinogammarus marinus* is a key-species from European estuarine habitats with a distribution ranging from Norway to Portugal [1]. The present article contains supportive data related to a research article entitled ‘Comparing production and life-history traits of a key amphipod species within and between estuaries under different levels of anthropogenic pressure’ [2]. The present dataset presents the density, biomass, fecundity, and production of *E. marinus* in three estuaries under different anthropogenic pressure and, within each estuary, at three sampling sites, which differed in terms of the distance to the estuary mouth, vegetation cover, and organic matter content. Monthly environmental abiotic data and seasonal concentration of PAH and other contaminants are also provided. Sampling took place monthly for 13 months at low tide on intertidal mudflats. At each site, *Fucus* fronds containing *E. marinus* individuals were randomly collected. All *E. marinus* individuals were counted, sexed, and measured under a binocular stereo microscope to estimate the density and the biomass of *E. marinus* in *Fucus* fronds. Finally, the annual production of *E. marinus* at each sampling site was estimated through the size-frequency method. This dataset may be used to compare population traits of *E. marinus* populations across different estuaries and it may overall assist designing studies regarding population dynamics and designing management strategies in coastal systems, namely targeting at habitat conservation and restoration.

**Specifications Table**

**Table:** 

Subject	Environmental Sciences (Ecology)
Specific subject area	Macroinvertebrate population dynamics and production in estuaries
Type of data	Figures Tables
How the data were acquired	Monthly low tide field sampling of *E. marinus* individuals associated with intertidal *Fucus* fronds and the underneath sediment (3 replicates of *Fucus* spp. fronds containing *E. marinus* per sampling site), along with records of physico-chemical parameters and collection of water and sediment samples between July 2011 and August 2012. Environmental data (physico-chemical parameters: water temperature, salinity, dissolved oxygen, pH, oxidation-reduction potential (ORP)) measured *in situ* with YSI multiparameter probe. Organic matter content of the sediment obtained through standard methods. Nutrient (nitrate, nitrite, ammonia, phosphate) analyses with the segmented continuous flow multiparameter analyser Skalar® San++System Autoanalyzer. Seasonal contaminant (Cd, Cu, PAH) concentrations determined through standard methods by external labs. Assessment of the fresh weight, dry weight and ash-free dry weight (AFDW) of *Fucus* in the laboratory, and estimation of *Fucus* fronds cover area using ImageJ software in photographs obtained at the sampling sites. *E. marinus* cephalic length measured to the nearest 0.02mm under a binocular stereo microscope with a calibrated graduate ocular. Sex identification and egg counts made under a binocular stereo microscope.
Data format	Raw Analyzed Filtered
Description of data collection	Monthly data on *E. marinus* individuals associated with *Fucus* fronds, physico-chemical parameters and seasonal contaminants, between July 2011 and August 2012. Data obtained in three estuaries with different anthropogenic pressure (Minho<Mondego<Ave); three sampling sites within each estuary chosen according to distance to estuary mouth, vegetal cover and organic matter content.
Data source location	City/Town/Region: Caminha (Minho estuary), Vila do Conde (Ave estuary) and Figueira da Foz (Mondego estuary) Country: Portugal Latitude and Longitude for the sampling sites: Minho estuary EMB- 41°52′00″N, 8°51′02″W INT- 41°52′14″N, 8°50′41″W SAP- 41°52′45″N, 8°50′41″W Ave estuary PRA- 41°20′33″N, 8°44′43″W CAN- 41°20′26″N, 8°44′44″W EST- 41°20′30″N, 8°44′39″W Mondego estuary DOC- 40°08′34″N, 8°51′02″W ARM- 40°07′17″N, 8°50′29″W PRT- 40°07′07″N, 8°49′48″W
Data accessibility	Repository name: Life-history data of Echinogammarus marinus from three NE Atlantic estuaries under different levels of anthropogenic pressure (Mendeley Data) DOI: http://dx.doi.org/10.17632/cpszj6bc6p.1 Direct link to the dataset: https://data.mendeley.com/datasets/cpszj6bc6p/1
Related research article	Martins, I., Guerra, A., Leite, N., Constantino, E., Ilarri, M.I., Souza, A.T., Santos, M.M., Ford, A.T., Campos, J., 2022. Comparing production and life-history traits of a key amphipod species within and between estuaries under different levels of anthropogenic pressure. Marine Environmental Research 173, 105538. https://doi.org/10.1016/j.marenvres.2021.105538.

## Value of the Data

• The dataset provides information on the monthly fluctuations of *E. marinus* life-history features in 3 estuaries with differing contaminant levels, during an annual cycle.
• The data can be used by scientists, government agencies dedicated to the management of aquatic habitats, ONGs with interest in estuarine ecosystems.
• The dataset can be used to uncover growth and reproduction patterns, for future comparisons with other populations under different environmental pressures, and for modelling purposes.

## 1 Data Description

Here we present metadata containing abiotic and biotic parameters related with the amphipod *E. marinus* obtained at three different estuaries, and within each estuary at three different sites, along the North and Central coast of Portugal for the period between July 2011 and August 2012. This metadata is presented in two different files and each file contains several tables. File “Data_Abiotic_Biotic.xlsx” contains four tables with data taken for each site and estuary: 1- “FQ & Contaminants” presents the monthly observations of water temperature, salinity, dissolved oxygen (DO), oxygen-reduction potential (ORP), organic matter content of the sediment, and dissolved nutrients (P-PO_4_, N-NO_2_, N-NO_3_, N-NH_4_); it also contains the seasonal values of the contaminants Cu, Cd and tPAH. 2- “*E. marinus* density” presents the monthly values of *Fucus* weight and density, number of *E. marinus* and density per population group: juveniles, immature females, adult females and males. 3- “*E. marinus* Biomass'' presents *E. marinus* total biomass and biomass per population group and sampling month. 4- “*E. marinus* Fecundity” presents the monthly values of the number of females with eggs, the number of eggs and number of eggs per female (fecundity).

File “Data_Production.xlsx” contains two tables. 1- “Densities (Yj) & Total Days (D) presents per estuary, site, and month, the number of days between samplings, the total number of days and *E. marinus* density per size class. 2- “Annual Production” presents per estuary, site, and size class, the estimation of the parameters to obtain the annual production. It also presents, for each site, the number of size classes, the cohort production interval (CPI), the total production, the annual production, the annual biomass and the P/B.

**Fig. 1:**
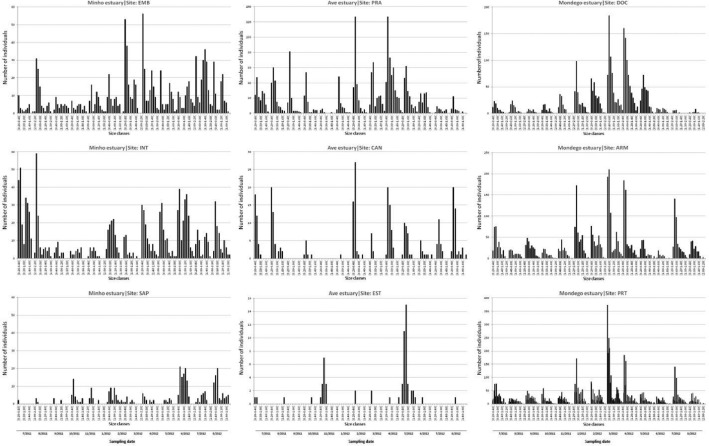
Size-frequency distributions of *Echinogammarus marinus* at each sampling site for the period between July 2011 and August 2012.

The number of *E. marinus* per size class at each site over time (histograms) is presented in the Fig. 1.

## 2 Experimental Design, Materials and Methods

### 2.1 Experimental design

A 13-month intertidal sampling program (13 × 3 × 3 × 3, respectively months, estuaries, sites, and replicates) was conducted monthly at three Portuguese river estuaries with different levels of anthropogenic impact: Minho<Mondego<Ave. At each estuary three sites were sampled during low water. Sites were chosen according to distance to the estuary mouth, organic matter content of the sediment and human-induced disturbance, thus, covering different habitats within each estuary. At each site, three *Fucus* spp. fronds replicates containing *E. marinus* were randomly chosen and sampled along with 5 cm of the underneath sediment. Physico-chemical parameters were measured in situ, and water and sediment samples were collected for posterior analysis of dissolved nutrient concentrations and organic matter content, respectively. Seasonal sediment samples were collected for determination of contaminant concentration. The selection of contaminants was determined by the probability of their occurrence in estuarine sediments.

### 2.2 Study sites

The three studied estuaries are located on the NW coast of Portugal with a distribution from north to south corresponding to Minho, Ave and Mondego.

The Minho river is an international river flowing across the NW regions of Spain and Portugal estuary. Nevertheless, the Minho river estuary has been described as containing lower levels of chemical contamination compared to other Portuguese estuaries [3] and it has also been classified with ‘Good’ ecological status (according to the metrics of the Water Framework Directive 2000/60/EC, WFD), based on fish [4] and phytoplankton assemblages [5]. Within the Minho estuary, EMB is located close to the estuary mouth on a sand-muddy area (935 m upstream), INT is located further upstream on a sand-muddy area (1532 m) at a small fishing harbor and SAP is located further upstream (4029 m) on a muddy area corresponding to a seagrass bed.

The Ave river is highly impacted mainly by agriculture and industrial activities [6] and is considered one the most polluted rivers in Portugal [7]. At the estuary, CAN is a rocky area located about 188 m upstream at the margins of the main river channel, the sampled site PRA corresponds to a sand area located at 352 m upstream from the estuary mouth, and EST is a mud area and location of an abandoned boatyard (443 m upstream).

The Mondego estuary is located in the central region of Portugal and considered moderately contaminated [8]. However, due to nutrient runoff from the upstream rice fields, this estuary tends to be strongly eutrophic and, as a consequence, seasonal macroalgal blooms tend to occur [9,10]. Nonetheless, this situation has improved in the last two decades due to the application of mitigation measures including adequate water management [11]. The site DOC is a muddy sand area located at 1384 m upstream from the estuary mouth, ARM is a muddy sand area located further upstream (3967 m) and PRT is a muddy area located at about 5091 m upstream the estuary mouth.

### 2.3 Sampling and analysis of abiotic parameters

In each monthly campaign, water temperature, salinity, dissolved oxygen, pH and oxidation-reduction potential (ORP)) were measured in situ with a multiparameter sonde (YSI Professional Plus). *In situ* water samples for nutrient analysis were collected to plastic bottles and kept inside cooling containers. Upon arrival at the laboratory, water samples were filtered using a vacuum pump and Whatman GF/F glass-fibre filters. The filtered samples were stored in 40 ml vials at -18°C and later analyzed for nutrient content using the Skalar® San++System Autoanalyzer, a segmented continuous flow multiparameter analyser. Concentrations are given in mgl^−1^. Sediment samples were collected and kept inside plastic bags in a cooling container. In the lab, these samples were placed in paper boxes (10 × 10 × 5 cm) and left to dry on a stove at 60°C for at least 72 h. Afterwards the samples were macerated into ceramic crucibles and burned in a muffle at 450°C for 8h to determine the ash-free dry weight (AFDW). Seasonal sediment samples were collected for Cd, Cu and PAH determination. These samples were transported in cooling containers and stored at -18°C until further analyses using standard methods were conducted by external laboratories (IPMA and Quimiteste, Portugal).

### 2.4 *Fucus* sp. and *E. marinus* sampling and analysis

In each sampling campaign, 3 sets of *Fucus* fronds were randomly chosen at each sampling site and photographed for posterior area analysis. *E. marinus* individuals were sampled along with the associated *Fucus* fronds plus 5 cm of the underneath sediment. The collected samples were split into two fractions: *Fucus* and sediment. The sediment fraction, which was previously sieved in the field with a 0.5 mm mesh to remove the excess of sediment, was stored in plastic bags and preserved with 4% buffered formalin until further processing. The *Fucus* fraction, containing mostly *Fucus* sp. and *E. marinus*, was carefully washed with tap water to remove all *E. marinus* specimens upon arrival to the lab. These were then stored in 80% ethanol until further processing. *Fucus* sp. was weighted for fresh weight, and dried on a stove at 60°C for at least 72 h to obtain dry weight. Seventy six samples of *Fucus* were also burned in a muffle at 450°C for 8h to estimate the average AFDW of the algae. From the photographs obtained *in situ*, the *Fucus* area (m^2^) was determined with appropriate software (ImageJ). Posteriorly, *E. marinus* individuals were counted, sexed, and the cephalic length (CL) was measured to the nearest 0.02 mm under a binocular stereo microscope with a calibrated graduate ocular.

The determination of sex was based on the presence or absence of oostegites and/or broods (females), and of genital papillae (males). Animals without these features were considered juveniles; individuals with both features were classified as intersex males if they had genital papillae and rudimentary brood plates, or intersex females if the less developed character were the genital papillae [12]. Nonbreeding females were examined for the presence or absence of setae on the oostegites and classified as mature or immature (resting) females, considering as immature the females with a cephalic length shorter than the smallest ovigerous female found (females with CL < 1.02 mm) [13]. If broods were present, eggs were counted under a binocular stereo microscope to estimate fecundity.

Histograms of size-frequency distribution (Fig. 1) were constructed for each sampling site and sampling date to estimate the cohort production interval (CPI) as the mean life span of the different cohorts. In the present study, the estimated CPI was 366 days.

To estimate biomass, the mean individual ash-free dry weight (W) in each size class (Wj) was obtained from the allometric equation determined by [1]:

(1) $$W=1.592924\;\times CL^{3.94344}$$

where W refers to AFDW (g) and CL is the cephalic length (mm).

The density (Y) of *E. marinus* in the algae cover (indm^−2^) was related with both sampled area and algal density through the formula:

(2) Y=A×B

where, A is the number of amphipods per gram of algae in each sample, and B is the average weight of algae (g AFDW) per square meter per sampled date [1].

### 2.5 *E. marinus* annual production estimation

Finally, the annual secondary production (P) was estimated through the size-frequency method based on the Hynes average cohort method modified by [14] as described in [15]:

(3) $$P=\left[i\sum_{1}^{i}\left(Y_{j}-Y_{j+1}\right).\sqrt{\left(W_{j}.W_{j+1}\right)}\right].\frac{D}{CPI}$$

where i is the number of the size class (i = 1 to a), Yj is the mean density in size class j (indm^–2^), Wj is the mean individual weight in size class j (gAFDW), D is the sampling period (days) and CPI is the cohort production interval (days). The number of size classes (a) and the sampling period (D) differed between estuaries as follows: a = 99 size classes and D = 397 days in Minho; a = 89 size classes and D =393 days in Ave; and a = 109 size classes and D =394 days in Mondego.

## Ethics Statement

Not applicable.

## CRediT Author Statement

**Irene Martins:** Conceptualization; Funding acquisition; Investigation; Methodology; Project administration; Resources; Supervision; Writing – review & editing; **Alexandra Guerra:** Data curation; Methodology; Production calculation; Formal analysis; Investigation; **Nuno Leite** and **Emanuel Constantino:** Field and lab work; Data curation; Investigation; **Martina Ilarri** and **Allan T. Souza:** Formal analysis; Graphical work; Software; **Miguel Santos** and **Alex T. Ford:** Writing - editing & reviewing; **Joana Campos:** Conceptualization of the final manuscript; Writing, review & editing

## Declaration of Competing Interest

The authors declare that they have no known competing financial interests or personal relationships that could have appeared to influence the work reported in this paper.

## References

[1] Marques J.C., Nogueira A. (1991). Life cycle, dynamics and production of *Echinogammarus marinus* (Leach; Amphipoda) in the Mondego estuary (Portugal). Oceanol. Acta.

[2] Martins I., Guerra A., Leite N., Constantino E., Ilarri M.I., Souza A.T., Santos M.M., Ford A.T., Campos J. (2022). Comparing production and life-history traits of a key amphipod species within and between estuaries under different levels of anthropogenic pressure. Mar. Environ. Res..

[3] Capela R., Raimundo J., Santos M.M., Caetano M., Micaelo C., Vale C. (2016). The use of biomarkers as integrative tools for transitional water bodies monitoring in the Water Framework Directive context – a holistic approach in Minho river transitional waters. Sci. Total Environ..

[4] Cabral H., Fonseca V., Gamito R., Gonçalves C., Costa J., Erzinib K., Gonçalves J., Martins J., Leite L., Andrade J., Ramos S., Bordalo A., Amorim E., Neto J., Marques J., Rebelo J., Silva C., Castro N., Almeida P., Domingos I., Gordo L., Costa M. (2012). Ecological quality assessment of transitional waters based on fish assemblages in Portuguese estuaries: the Estuarine Fish Assessment Index (EFAI). Ecol. Indic..

[5] Brito A., Brotas V., Caetano M., Coutinho T., Bordalo A., Icely J., Neto J., Serôdio J., Moita T. (2012). Defining phytoplankton class boundaries in Portuguese transitional waters: an evaluation of the ecological quality status according to the water framework directive. Ecol. Indic..

[6] Araújo M.F., Valério P., Jouanneau J.M. (1998). Heavy metal assessment in sediments of the ave river basin (Portugal) by energy-dispersive x-ray fluorescence spectrometry. Spectrometry.

[7] Ribeiro C.M.R., Maia A.S., Ribeiro A.R., Couto C., Almeida A.A., Santos M., Tritan M.E. (2016). Anthropogenic pressure in a Portuguese river: endocrine-disrupting compounds, trace elements and nutrients. J. Environ. Sci. Health, Part A.

[8] Caetano M., Raimundo J., Nogueira M., Santos M., Mil-Homens M., Prego R., Vale C. (2016). Defining benchmark values for nutrients under the water framework directive: application in twelve Portuguese estuaries. Mar. Chem..

[9] Lillebø A.I., Neto J.M., Martins I., Verdelhos T., Leston S., Cardoso P.G., Ferreira S.M., Marques J.C., Pardal M.A. (2005). Management of a shallow temperate estuary to control eutrophication: the effect of hydrodynamics on the system nutrient loading. Estuar. Coast. Shelf Sci..

[10] Martins I., Lopes R.J., Lillebø A.I., Neto J.M., Pardal M.A., Ferreira J.G., Marques J.C. (2007). Significant variations in the productivity of green macroalgae in a mesotidal estuary: Implications to the nutrient loading of the system and the adjacent coastal area. Mar. Pollut. Bull..

[11] Dolbeth M., Cardoso P.G., Grilo T.F., Bordalo M.D., Raffaelli D., Pardal M.A. (2011). Long-term changes in the production by estuarine macrobenthos affected by multiple stressors. Estuar. Coast. Shelf Sci..

[12] Ford A.T., Fernandes T.F., Rider S.A., Read P.A., Robinson C.D., Davies I.M. (2003). Reproduction in the amphipod, *Echinogammarus marinus*: a comparison between normal and intersex specimens. J. Mar. Biol. Assoc. U. K..

[13] Maranhão P., Bengala N., Pardal M., Marques J.C. (2001). The influence of environmental factors on the population dynamics, reproductive biology and productivity of *Echinogammarus marinus* Leach (Amphipoda, Gammaridae) in the Mondego estuary (Portugal). Acta Oecol..

[14] Benke A.C. (1979). A modification of the Hynes method for estimating secondary production with particular significance for multivoltine populations. Limnol. Oceanogr..

[15] Menzie C.A. (1980). A note on the Hynes method of estimating secondary production. Limnol. Oceanogr..

